# *In vivo* interspecies dissemination of IncM2-type *bla*_NDM-1_ carrying plasmid

**DOI:** 10.1128/spectrum.00399-24

**Published:** 2024-12-17

**Authors:** Mor N. Lurie-Weinberger, Darya Bychenko-Banyas, Meirav Mor, Sivan Laviad-Shitrit, Ella Kaplan, Nadya Rakovitsky, Alona Keren-Paz, Chaim Ben-Zvi, Yehuda Carmeli

**Affiliations:** 1 National Institute for Antibiotic Resistance and Infection Control, Ministry of Health, Tel Aviv, Israel; 2 Schneider Children’s Medical Center of Israel, Petach Tikva, Israel; 3 Faculty of Medicine, Tel Aviv University 26745 , Tel Aviv-Yafo, Israel; 4 Microbiology Laboratory, Rabin Medical Center, Beilinson Hospital, Petach Tikva, Israel; Michigan State University, East Lansing, Michigan, USA

**Keywords:** antibiotic resistance, evolution, plasmids

## Abstract

The gastrointestinal tract is a unique ecological niche with a high abundance of various Enterobacterales in close proximity. This allows the exchange of mobile genetic elements that carry resistance determinants. In the hospital setting, resistant organisms are prevalent and selective antibiotic pressure is high, providing a supportive platform for interspecies dissemination of resistance. Seven New Delhi metallo-beta-lactamase(NDM) positive and one NDM-negative bacteria were sent for WGS followed by bioinformatics analysis. Here, we describe three separate cases of patients simultaneously colonized by two NDM producing-species (*Escherichia coli* and *Klebsiella pneumoniae*), in which we documented interspecies dissemination of the *bla*_NDM-1_ by an 87,450 bp IncM2 type multi-drug resistance plasmid (here named pNCICAN_NDM-01_21-il). This plasmid was found to be highly conjugative. Our results underline the risk of interspecies horizontal plasmid dissemination. Such events may cause the emergence of high-risk clones, as well as lead to under-recognized multi-species outbreaks.

**IMPORTANCE:**

Conjugative, carbapenemase-carrying multidrug-resistant plasmids that can move between species of clinically relevant Enterobacterales pose a great risk to patients’ health, especially when they spread inside a medical institution. Yet, most institutions monitor bacteria according to species and are at risk of missing plasmid-driven outbreaks. Thus, this work indicates that plasmid surveillance is an important tool for infection control.

## INTRODUCTION

In a 2014 CDC paper summarizing a multistate point prevalence survey of healthcare-associated infections, the most commonly isolated pathogens causing nosocomial infections were *Escherichia coli* (9.3%) and *Klebsiella pneumoniae* (9.9%) (1). In recent years, with the rise in multidrug-resistant bacteria worldwide, resistant pathogens of the order Enterobacterales have become an increasing threat to human health (2).

A human patient gut is, *de facto*, an ecological niche, where bacteria of different species co-exist (3). The proximity of bacterial species in a given anatomic site area provides an opportunity for gene exchange (3, 4). As antibiotic exposure creates selective pressure and organisms’ acquisition of resistance mechanisms by lateral gene transfer provides an evolutionary advantage, the spread of multidrug-resistant plasmids becomes a distinct possibility. There are some recent reports regarding the pervasive transmission of OXA48 (5) and KPC (6) carrying plasmids in the gut microbiota of hospitalized patients, yet these events are not frequently reported.

Here, we describe a series of three cases of patients co-colonized by two Enterobacterales species with the same carbapenemase-carrying multidrug-resistant plasmid in a clinical setting, suggesting *in vivo* interspecies plasmid dissemination.

## RESULTS

### Screening of patients for carbapenemase-producing bacteria

Over an 8-day period in late 2021 (Table S1), three patients in a tertiary pediatric hospital in Israel were found to simultaneously carry NDM-positive *E. coli* and NDM-positive *K. pneumoniae*. All patients had at least one isolate of each species. In addition, a carbapenem-sensitive *E. coli* isolate from patient 1 was available. An epidemiological investigation revealed that one patient had been admitted 15 days prior to screening and had tested negative for carrying carbapenem-resistant enterobacteriaceae (CPE) upon admission, while the other patients had not been previously screened.

### Antibiotic susceptibility

**Fig 1:**
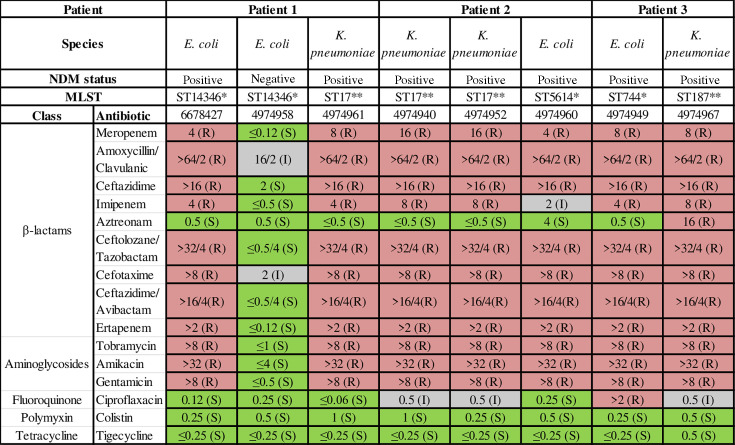
Antibiotic-susceptibility profile by broth microdilution of all isolates in this study. *As per the Achtman scheme. **As per the Pasteur MLST scheme. ^a^Clavulanic acid at a fixed concentration of 2 µg/mL. ^b^Tazobactam and avibactam at a fixed concentration of 4 µg/mL.

These seven isolates, four *K*. *pneumoniae* and three *E. coli* had a nearly uniform resistance profile(Fig. 1). They were resistant to β-lactams (including ertapenem and meropenem), as well as gentamicin, colistin, and tetracycline. Six of the seven were also resistant to ciprofloxacin. From one patient (patient 1), a non-NDM-producing *E. coli* was also available for further testing. This *E. coli* (isolate 4974958) was sensitive to meropenem, ertapenem, amikacin, and gentamicin (Fig. 1).

### WGS analysis

To further understand the relationship between the *E. coli* and *K. pneumoniae* isolates, the complete genome sequence of all eight isolates was determined using Illumina sequencing. Three of the four *K. pneumoniae* isolates were members of ST17, while the fourth was ST187, a sequence type that shares only *pgi* allele 1 and *rpoB* allele 4 with ST17. To further determine how closely related these isolates are, we analyzed them using genomic tools. The single nucleotide polymorphism (SNP) analysis shows that 6678427 and 4974958 differed by only 19 SNPs, while all other *E. coli* isolates were different from both 6678427 and each other by thousands of SNPs (Table S2).

In *E. coli*, each patient’s isolate had a unique multilocus sequence type (MLST) according to the Achtman scheme. The NDM-positive and the NDM-negative isolates from patient 1 belonged to the same ST (Fig. 1). The SNP analysis shows that isolates 4974940 and 4974952 were most closely related, differing only by 32 SNPs, while isolate 4974961 was more similar to isolates 4974940 and 4974952 (with 331 and 339 SNPs, respectively). The last isolate, 4974967, was most different from all other isolates, with thousands of SNPs difference (Table S3).

**Fig 2:**
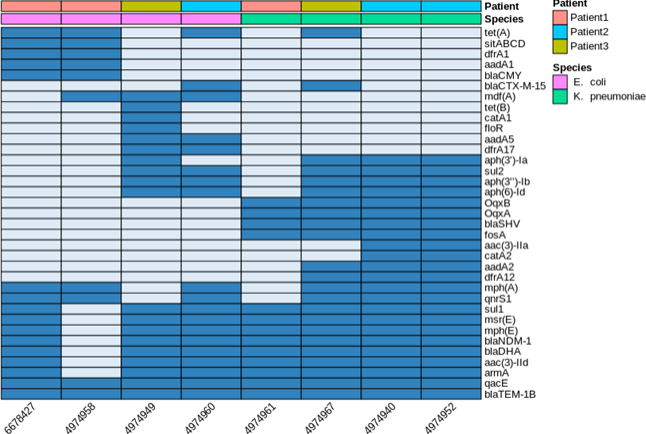
Antibiotic-resistance genes found in the study isolates. Blue indicates gene presence, and gray indicates gene absence.

All seven NDM-positive isolates carried a large array of antibiotic-resistance genes including *sul1*, *armA, blaDHA, mph(E), msr(E)*, and the antiseptic-resistance gene *qacE*, but differed in the pattern of the ARG array (Fig. 2). Plasmid content indicated the presence of several incompatibility type *rep* genes in the respective genomes (Table S1). All NDM-positive isolates contained IncM2, while the NDM-negative *E. coli* did not.

### IncM2-type plasmid

**Fig 3:**
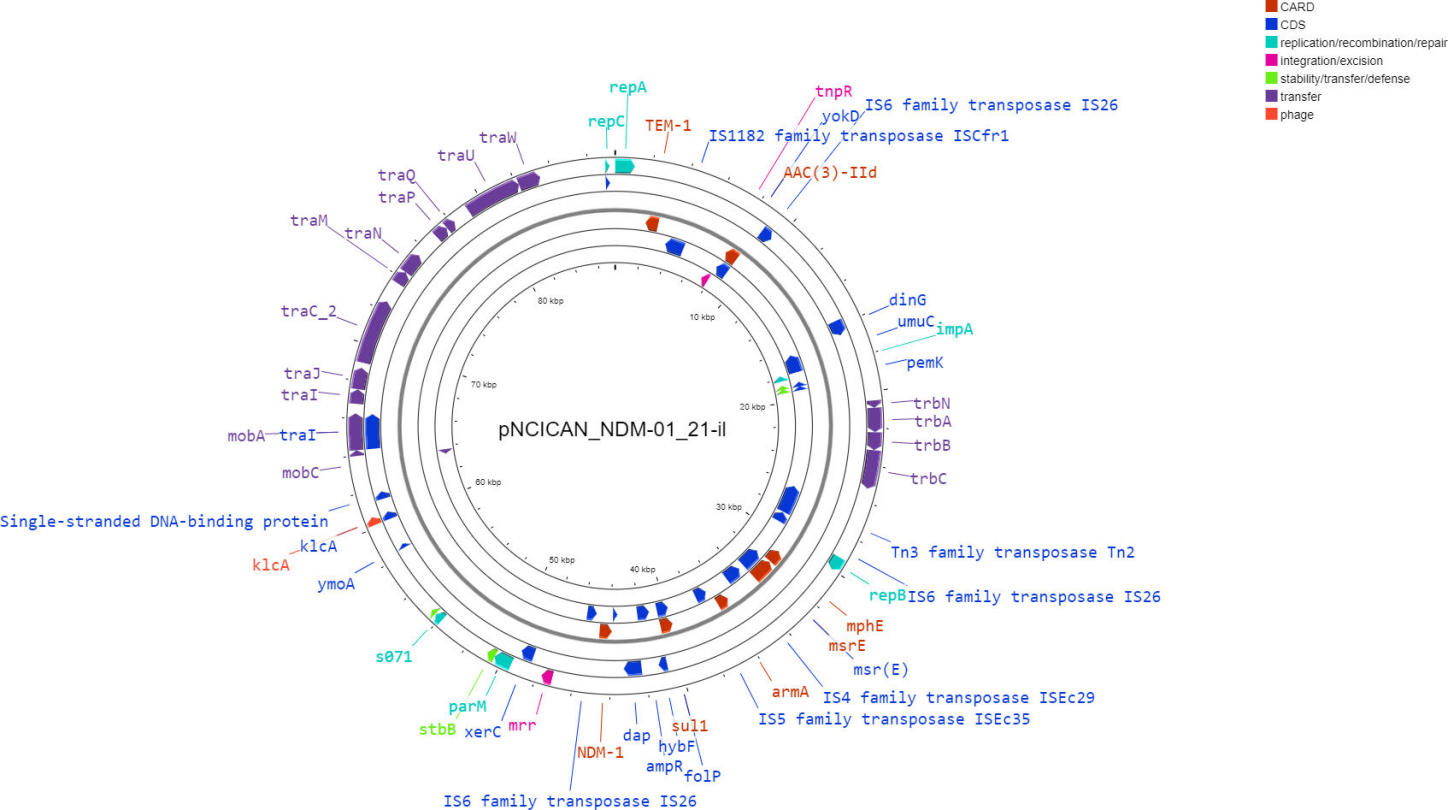
The pNCICAN_NDM-01_21-il plasmid.

To obtain a full-length sequence of the putative IncM2 plasmid, long-read sequencing using Oxford Nanopore and short-read Illumina sequencing were performed on *E. coli* isolate 4974960, and a composite assembly was constructed using both types of reads. We verified the presence of this plasmid in all seven isolates by Oxford Nanopore sequencing. The plasmid was an 87,450 bp long IncM2 type plasmid with both repA and repB (Fig. 3). It carried the carbapenemase *bla*_NDM-1_, as well as *bla_TEM-1B_, mph(E), msr(E), acc (3)-IId, armA, sul1,* and DHA-7. The content of all plasmids was 100% identical (as per blast2seq). Six mobile elements were present across the plasmid: IS1182 family transposase ISCfr1, IS6 family transposase IS26, IS6 family transposase IS15, IS4 family transposase ISEc29, IS5 family transposase ISEc35, IS6 family transposase IS26, and Tn3 family transposase Tn2. *bla*_NDM-1_ , *sul1, dap, ampR,* and *hybF* were flanked by IS6 family transposase IS26 and IS5 family transposase ISEc35. The genes *mph(E), msr(E)*, and *armA* were located directly downstream of the IS5 family transposase ISEc35 and upstream of the IS6 family transposase IS15.

The plasmid backbone contained the pilus-related *traA, traB, traC,* and *traW,* transcription factor *traJ,* the relaxosome *traM, traN* (mating pair stabilization)*, traU* (DNA transfer), as well as mobilization genes *mobA* and *mobB* (Fig. 3). It also contained the programmed cell death toxin PemK and the antitoxin PemI. We named this plasmid pNCICAN_NDM-01_21-il.

A blastN search of the study plasmid found that similar plasmids have been previously described in other *K. pneumoniae* isolates (LC536683.1 that has 99.98% identity over 92% length of the plasmid), *E. coli* (HQ451074.1, 99.99% similarity over 100% length), and *Salmonella enterica* (CP032193.1, 100% similarity over 100% length). This suggests that the plasmid is transferable between species. The first plasmid to be deposited into NCBI was an *E. coli* plasmid, entered in 2010, followed by an *S. enterica* plasmid in 2018, and finally by a *K. pneumoniae* plasmid in 2020. The study plasmid was detected late in 2021. These plasmids were found in a wide range of geographical locations including Japan (LC536683.1), China (HQ451074.1), and the United States (CP032193.1), indicating a worldwide spread of this plasmid. Those three plasmids are IncM2-type plasmids that carry the *blaNDM-1* allele, as well as *armA, blaDHA-7, blaNDM-1, mph(E), msr(E), qacE,* and *sul1*. HQ451074.1 and CP032193.1 carry *aac (3)-IId* and *blaTEM-1B*, while LC536683.1 does not. Further multiple alignment analysis shows that our plasmid and LC536683.1 are more closely related to one another than to the two additional plasmids (Fig. S1).

### Conjugation assay

To test conjugation, we tested both transfers of the IncM2 plasmid from a *K. pneumoniae* donor into an *E.coli* recipient and vice versa. Conjugation frequency (transconjugants/donor CFU) between *K. pneumoniae* isolate 4974961 into *E.coli* DH10β with a pHSG396 plasmid (conferring chloramphenicol resistance) was 2 × 10^−5^. Similarly, conjugation rates from *E. coli* isolate 4974960 into *K. pneumoniae* ATCC 700603 with pHSG396 was 6.4 × 10^−5^. These mating experiments yielded transconjugants that were resistant to carbapenems and carried the *blaNDM-1* gene, confirmed by PCR. Finally, a direct conjugation was performed between the NDM-positive *K. pneumoniae* isolate 4974961 from patient 1 to the NDM-negative *E.coli* isolate 4974958 (with added pHSG396 plasmid) from the same patient, resulting in an average conjugation rate of 6.64 × 10^−4^.

## DISCUSSION

Bacteria have the unique ability to acquire new genetic determinants from other bacterial species, thereby accelerating their evolution (7). Previous studies have estimated that significant portions of the Open Reading Frames in all fully sequenced genomes are the result of lateral gene transfer (8, 9). This ability plays a pivotal role in the dissemination of antibiotic-resistance determinants. The combination of antibiotic pressure and the presence of mobile elements carrying antibiotic-resistance genes greatly increases the chances for the transfer of the element into new susceptible bacteria (3).

In this study, we described three cases of patient co-colonization by *E. coli* and *K. pneumoniae*, where both bacteria harbored the same IncM2 plasmid. This shared plasmid carried various antibiotic-resistance genes (ARGs): *bla*_NDM-1_, *bla*_TEM-1B_, *mph(E), msr(E), acc (3)-IId, armA, sul1,* and DHA-7. The plasmid appeared to be highly transferable, as it harbored a set of *tra, mob,* and *rep* genes enabling its mobility. We experimentally verified that transfer by conjugation is indeed possible between species, with a conjugation rate of 2 × 10^−5^ from *K. pneumoniae* to *E. coli* and reciprocal conjugation from *E. coli into K. pneumoniae,* 6.4 × 10^−5^. We have also performed a direct conjugation between the NDM-positive *K. pneumoniae* isolate 4974961 from patient 1 and the NDM-negative *E. coli* isolate 4974958 from the same patient, resulting in a higher average conjugation rate of 6.64 × 10^−4^. The conjugants gained resistance to meropenem and ertapenem, indicating that the plasmid can pass between, and confer resistance to, both species. In one patient, we found two isogenic *E.coli* strains, one carrying the IncM NDM-encoding plasmid, and one—without it. The strain lacking the plasmid likely represents the “parent” susceptible strain, that later gained the plasmid. However, plasmid loss in a previously NDM-positive strain is also possible. We determined that while 3/4 *K*. *pneumoniae* belonged to the same ST type and all were closely related, the *E. coli* isolates carrying the plasmid represented distinct STs. This, together with the presence of two almost identical *E. coli* isolates in a single patient, indicates that the more likely scenario is that the plasmid originated in *K. pneumoniae* and was transferred into *E. coli* on several distinct occasions. As conjugation events are sporadic, not all *E. coli* isolates were able to attain the plasmid, resulting in a mosaic pattern of both NDM-positive and NDM-negative *E. coli* in a single patient.

The IncM family in general, and the IncM2 type in particular, are known to be clinically important and are abundantly found in public databases (10). They carry an array of antibiotic-resistance genes including the β-lactamase *bla*_NDM_ as well as the macrolide phosphotransferase *mph*(E) and the sulfonamide-resistance gene *sul1* (10). This underlines the importance of IncM2 plasmids in the dissemination of antibiotic-resistance genes, including carbapenemases (10, 11). There are six major resistance plasmid families identified in clinically relevant Enterobacterales (11). Of these, IncL/M plasmids, and specifically IncM2 *bla*_NDM-1_ positive plasmids, have been extensively characterized (10, 11) and are known to play a role in the dissemination of *bla*_NDM-1_. Moreover, IncM plasmids are thought to constitute an important inter-species vehicle for the spread of resistance genes (11). IncM2 plasmids carrying *bla*_NDM-1_ were described in at least five different species of Enterobacterales (including *E. coli* and *K. pneumoniae*) (12), suggesting high transferability. In a sequential report, the conjugation efficiency of these IncM2 plasmids from *K. pneumoniae* donor to *E. coli* recipient was 10^−3^ – 10^−5^ (13), comparable with the rate of 10^−5^ reported here (6).

Patients in clinical settings constitute a unique environmental niche. The host is often more vulnerable to infection than a healthy individual, allowing for co-infection due to the proximity of different bacteria over an extended period of time. Moreover, the patient often receives antibiotics, creating selective pressure for lateral gene transfer (4). All these conditions favor the transfer of mobile genetic elements, such as plasmids, between pathogens (4). Yet, most infection detection paradigms concentrate on species; if a single patient carries two antibiotic-resistant bacteria, each infection is considered separately, obscuring the extent of plasmid-driven outbreaks. For successful infection control and a better understanding of outbreaks, it is crucial to monitor plasmid dissemination and not just bacterial species, as plasmids can be incorporated into significantly different genomic contexts, drastically expanding the array of bacteria with limited treatment options.

## MATERIALS AND METHODS

### Isolates

Samples were obtained by rectal swabs during routine surveillance of carbapenemase-resistant Enterobacterales in the Israeli hospital. Colonies growing on selective chromogenic media (CHROMagar^™^ mSuperCarba^™^, HyLabs, Rehovot, Israel) were identified by VITEK-MS^®^ MALDI-ToF (Bruker Daltonics). CPE isolates were transferred to the National Institute for Antibiotic Resistance and Infection Control for further analysis.

### Antibiotic susceptibly testing

MICs were determined by broth microdilution (Sensititre^™^ Gram Negative DKMGN Kit, ThermoFisher Scientific, Oakwood Village, OH, USA) according to manufacturer’s instructions and interpreted according to the Clinical and Laboratory Standards Institute (CLSI) M100 guidelines (14).

### WGS and bioinformatics analysis

ST typing was determined using https://pubmlst.org using the Pasteur scheme for *K. pneumoniae* isolates and the Achtman scheme for *E. coli*. Antibiotic-resistance genes were indicated by ResFinder (https://cge.food.dtu.dk/services/ResFinder/) (15). Plasmids were detected with https://cge.food.dtu.dk/services/PlasmidFinder/ (16). Plasmid assembly was performed using Unicycler v.0.4.8 with both long and short reads. Plasmids were compared to one another using blast2seq to determine identity and coverage percentage. The identification of acquired virulence genes was performed by https://cge.food.dtu.dk/services/VirulenceFinder/. ANI calculations were performed using https://www.ezbiocloud.net/tools/ani/ (17). GGDC calculations were done with the Genome-to-Genome Distance Calculator (https://ggdc.dsmz.de/ggdc.php) (18). Plasmid annotation and visualization were performed with Proksee (19). The multiple alignment of plasmids was done using MAFFT and a phylogenetic tree was constructed using NJ. The SNP analysis was performed with Snippy version 4.6.0 (20).

### Conjugation assay

A filter mating conjugation was performed according to the protocol described previously (21) using the Millipore manifold system, with *K. pneumoniae* 4974961 as donor and *E. coli* strain DH10β with the pHSG396 plasmid (for chloramphenicol resistance) as recipient, and *E. coli* 4974960 as a donor with *K. pneumoniae* ATCC 700603 strain with the pHSG396 plasmid as a recipient, at a ratio of 1:2. Transconjugants were selected on Luria broth (LB) supplemented with 13 mg/L chloramphenicol and 0.5 mg/L meropenem, and further confirmed as NDM-positive (22) by PCR. Conjugation frequencies were calculated as the number of transconjugants (CFU/mL) obtained per donor (CFU/mL). All experiments were performed in three biological repeats, each in a technical triplicate. Conjugants’ antibiotic resistance was tested with VITEK. In addition, a similar experiment was performed using *K. pneumoniae* 4974961 as a donor and *E. coli* 4974958 with the pHSG396 plasmid as a recipient, both originating from patient 1.

## Data Availability

Whole genome assemblies are available under BioProject PRJNA1043582. The assembled plasmid of isolate 4974960 was deposited to BankIt under accession number 2871503.

## SUPPLEMENTAL MATERIAL

Supplemental material spectrum.00399-24-s0001.docx Tables S1 to S4; Fig. S1.

ASM does not own the copyrights to Supplemental Material that may be linked to, or accessed through, an article. The authors have granted ASM a non-exclusive, world-wide license to publish the Supplemental Material files. Please contact the corresponding author directly for reuse.
