# Supplementary material for: In vivo interspecies dissemination of IncM2-type blaNDM-1 carrying plasmid
Source: Microbiol Spectr. 2024 Dec 17;13(2):e00399-24. doi: 10.1128/spectrum.00399-24 (PMC11792519; doi:10.1128/spectrum.00399-24)
Supplement: Supplemental material — Tables S1 to S4; Fig. S1. [file spectrum.00399-24-s0001.docx]

**Supplementary Table 1: Characteristics of study isolates**

| Patient | ID | Date isolated | Species | NDM allele | Inc group |
| --- | --- | --- | --- | --- | --- |
| 1 | 6678427 | 22/10/2021 | *E. coli* | *bla*NDM-1 | IncB/O/K/Z, IncFII, IncM2 |
|  | 4974958 | 23/10/2021 | *E. coli* | *-* | IncFIB(K), IncFII |
|  | 4974961 | 23/10/2021 | *K. pneumoniae* | *bla*NDM-1 | IncFIB(K), IncFII, IncM2, IncR |
| 2 | 4974960 | 21/10/2021 | *E. coli* | *bla*NDM-1 | IncB/O/K/Z, IncM2 |
|  | 4974940 | 21/10/2021 | *K. pneumoniae* | *bla*NDM-1 | IncFIB(K), IncFII, IncM2, IncR |
|  | 4974952 | 21/10/2021 | *K. pneumoniae* | *bla*NDM-1 | IncFIB(K), IncFII, IncM2, IncR |
| 3 | 4974949 | 29/10/2021 | *E. coli* | *bla*NDM-1 | IncFII, IncM2 |
|  | 4974967 | 29/10/2021 | *K. pneumoniae* | *bla*NDM-1 | IncFIB(K), IncM2, IncR |

*As per the Achtman scheme. ** As per Pasteur MLST scheme.

**Supplementary table 2 - SNP distance between *E. coli* isolates**

|  | 6678427 | 4974949 | 4974958 | 4974960 |
| --- | --- | --- | --- | --- |
| 6678427 |  | 73086 | 19 | 76599 |
| 4974949 |  |  | 73415 | 42062 |
| 4974958 |  |  |  | 76298 |
| 4974960 |  |  |  |  |

**Supplementary table 3 - SNP distance between *K. pneumoniae* isolates**

|  | 4974940 | 4974952 | 4974961 | 4974967 |
| --- | --- | --- | --- | --- |
| 4974940 |  | 32 | 331 | 2774 |
| 4974952 |  |  | 390 | 27608 |
| 4974961 |  |  |  | 27638 |
| 4974967 |  |  |  |  |

**Supplementary Table 4: Virulence factors shared by NDM-positive and NDM-negative *E. coli* isolates from a single patient (isolates 6678427 and 4974958)**

| **Virulence factor** | **Protein function** |
| --- | --- |
| *air* | Enteroaggregative immunoglobulin repeat protein |
| *anr* | AraC negative regulator |
| *AslA* | Arylsulfatase |
| *chuA* | Outer membrane hemin receptor |
| *csgA* | Curlin major subunit CsgA |
| *eilA* | Salmonella HilA homolog |
| *espY2* | Effector |
| *fdeC* | Intimin-like adhesin FdeC |
| *fimH* | Type 1 fimbriae |
| *gad* | Glutamate decarboxylase |
| *hha* | Hemolysin expression modulator Hha (previous rmoA) |
| *hlyE* | Avian E. coli haemolysin |
| *iss* | Increased serum survival |
| *kpsE* | Capsule polysaccharide export inner-membrane protein |
| *kpsMIII_K96* | ABC-type polysaccharide/polyol phosphate export systems permease; Group 3 capsule |
| *lpfA* | Long polar fimbriae |
| *nlpI* | Lipoprotein NlpI precursor |
| *ompT* | Outer membrane protease (protein protease 7) |
| *sitA* | Iron transport protein |
| *terC_a* | Tellurium ion resistance protein |
| *terC_b* | Tellurium ion resistance protein |
| *traJ* | Protein TraJ (Positive regulator of conjugal transfer operon) |
| *traT_a* | Outer membrane protein complement resistance |
| *traT_b* | Outer membrane protein complement resistance |
| *yehB* | Usher, YHD fimbriael cluster |
| *yehC* | Chaperone, YHD fimbriael cluster |
| *yehD* | Major pilin subunit, YHD fimbriael cluster |


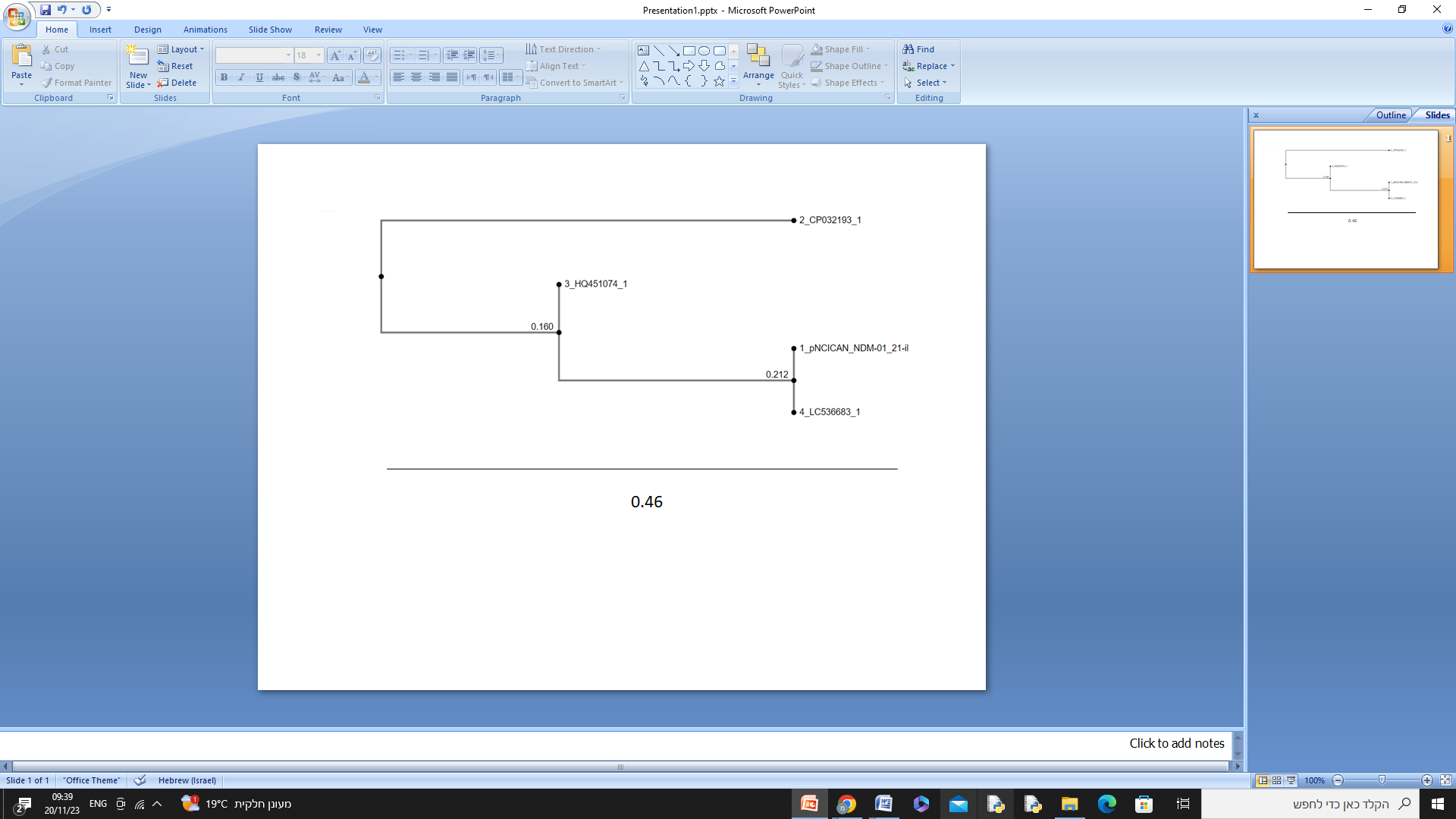


**Supplementary Figure 1: Phylogenetic tree of pNCICAN_NDM-01_21-il plasmid and its closest blastN hit in *K. pneumoniae,* *E. coli* and *S. enterica*.**
